# Exploring the Mechanism of Baicalin Intervention in Breast Cancer Based on MicroRNA Microarrays and Bioinformatics Strategies

**DOI:** 10.1155/2021/7624415

**Published:** 2021-12-20

**Authors:** Anqi Ge, Lifang Liu, Xian'guang Deng, Jun Luo, Yanghua Xu

**Affiliations:** ^1^The First Affiliated Hospital of Hunan University of Chinese Medicine, Changsha, Hunan, China; ^2^Hunan University of Chinese Medicine, Changsha, Hunan, China

## Abstract

**Objective:**

To explore the mechanism of baicalin intervention in breast cancer based on microRNA microarrays.

**Methods:**

The inhibitory rate of baicalin intervention in MCF-7 breast cancer cells was determined by MTT. Then, the miRNA microarrays were used to validate the key microRNAs. After that, reverse transcription-quantitative polymerase chain reaction (RT-qPCR) was used to validate microRNA, hsa-miR-15a, hsa-miR-100, hsa-miR-16, and hsa-miR-7t. Finally, the potential targets of these key microRNAs are predicted by miRWalk, and DAVID was utilized for gene ontology (GO) enrichment analysis and pathway enrichment analysis.

**Results:**

Baicalin may inhibit the proliferation of MCF-7 cells in a dose-dependent and time-dependent manner. The concentration of baicalin 150 *μ*mol/L was determined for the subsequent miRNA chip research. A total of 92 upregulated microRNAs and 35 downregulated microRNAs were obtained. The upregulated miRNAs include hsa-miR-6799-5p, hsa-miR-6126, hsa-miR-4792, hsa-miR-6848-5p, hsa-miR-3197, hsa-miR-6779-5p, and hsa-miR -654-5p. The downregulated miRNAs include hsa-miR-3911, hsa-miR-504-5p, hsa-miR-30a-3p, hsa-miR-193b-3p, and hsa-miR-181b-5p. Then, differentially expressed miRNA was verified by qRT-PCR. The results showed that the expression of hsa-miR-15a, hsa-miR-100, hsa-miR-16, and hsa-let-7c was upregulated (*P* < 0.05), which was consistent with the results of the miRNA microarray. The enrichment analysis showed that baicalin might regulate the DNA-templated proliferation, DNA-templated transcription, p53 signaling pathway, etc., of MCF-7 breast cancer cells through miRNA.

**Conclusion:**

Baicalin inhibits the proliferation of breast cancer cells. It may achieve antitumor effects through regulating microRNAs so as to affect the DNA replication (such as cellular response to DNA damage stimulus and DNA binding), RNA transcription (such as regulation of transcription, DNA-templated, transcription from RNA polymerase II promoter, and transcription factor binding), protein synthesis (such as mRNA binding, Golgi apparatus, and protein complex), endocytosis, pathways in cancer, p53 signaling pathway, and so on.

## 1. Introduction

As the global incidence of cancer rises sharply, breast cancer is one of the most common malignant tumors among women and has become the leading cause of cancer death in women. Worldwide, there are approximately 2.1 million newly diagnosed female breast cancer cases in 2018, accounting for a quarter of female cancers [1, 2]. Female breast cancer is the most common type of cancer in most countries (154/185) and is the leading cause of cancer deaths in more than 100 countries [3]. Breast cancer might result from multiple factors such as reproductive related factors, family accumulation and genetic factors, dietary factors, and breast disease history [4]. Breast cancer could be divided into four molecular subtypes based on molecular markers: luminal A type, luminal B type, triple-negative type, and HER2-overexpressive type [5, 6].

At present, the treatment of breast cancer is mainly based on surgical treatment and radiotherapy and chemotherapy, supplemented by targeted therapy and biological therapy. In adjuvant therapy, patients with luminal A- and luminal B-type breast cancer are mainly treated with endocrine therapy, and chemotherapy is mainly performed in patients with triple-negative breast cancer. In patients with HER2-overexpressing breast cancer, targeted therapy is used to target the HER2 gene [5, 6]. However, due to the toxic side effects and drug resistance of radiotherapy and chemotherapy, patients with tumor survival often have poor prognosis, making breast cancer treatment effect not ideal [7–9]. Therefore, finding and developing sensitizing drugs and antitumor drugs with good safety and low side effects have become the key to cancer treatment.

Baicalin is a flavonoid extracted from the dried roots of *Scutellaria baicalensis* Georgi. Current studies show that baicalin inhibits various biological behaviors of breast cancer, such as apoptosis, autophagy, epithelial-mesenchymal transition, migration, invasion, and metastasis, and enhances the sensitization of chemotherapy [10]. Its specific mechanism is regulation of the NF-*κ*B signaling pathway [11], Wnt/*β*-catenin signaling pathway [12], transforming growth factor (TGF)-*β* signaling pathway [13], AMPK/ULK1/mTOR signaling pathway [14], and ERK/p38MAPK signaling pathway [15]. However, the regulation mechanism of baicalin on breast cancer bionetworks still requires new strategies to discover. More importantly, recent studies have demonstrated that microRNAs play an important role in breast cancer bionetworks that regulate breast cancer proliferation, metastasis, and invasion by targeting downstream signaling pathways [16–18]. Therefore, this study integrated the microRNA microarrays and bioinformatics to discover the regulation mechanism of baicalin on breast cancer bionetworks. We also hope to provide a new paradigm for future research by designing a new strategy.

## 2. Materials and Methods

### 2.1. Experimental Drugs

Baicalin was purchased from China National Institute for the Control of Pharmaceutical and Biological Products, with a purity of 98.5% and product batch no. 120608-201113. The baicalin dry powder was fully dissolved in dimethyl sulfoxide (DMSO) and formulated into a stock solution with a concentration of 5000 *μ*mol/L, protected from light and stored at 4°C. The final concentration was 0 *μ*mol/L (NC), 50 *μ*mol/L, 100 *μ*mol/L, and 150 *μ*mol/L, diluted with the corresponding medium and DMSO.

### 2.2. Cell Lines of Breast Cancer and Normal Breast Cells

Human breast cancer cell line MCF-7 cells were purchased from the Cell Center of Xiangya School of Medicine, Central South University. Normal breast Hs 578Bst cell line was purchased from Shanghai Baili Biotechnology Co., Ltd. All were prepared by American Standard Biological Products Collection (ATCC).

The breast cancer cell line was cultured in the RPMI 1640 medium (containing 10% calf serum, penicillin 100 U/ml, and streptomycin 100 U/ml), cultured at 37°C, 100% humidity, 5% (volume fraction) CO_2_ incubator. Change the medium every 3 days. After the cells grow adherently, 0.25% trypsin is used for digestion and passage. Cells in the logarithmic growth phase were used for further experiments.

### 2.3. Reagents and Instruments

Penicillin/streptomycin (100x) and fetal bovine serum (FBS) were purchased from Sciencell Inc. D-Hank's solution, trypsin, and PBS buffer were obtained from the Central Laboratory of Xiangya Medical College. 0.05% trypsin + 0.38 mM EDTA was purchased from Gibco Inc. Cell culture plates were purchased from Costar Inc. Cell cryopreservation tube was purchased from China Biyuntian Biotechnology Research Institute. Agarose and DMSO were purchased from Gaylord Slidell Inc. Whole Transcriptome Amplification Kit (Real Time) Ver. 2 and real-time quantitative RT- PCR detection (qRT-PCR) kit were purchased from Bao Bioengineering (Dalian) Co., Ltd.; GoldVew nucleic acid dye was purchased from Shanghai Shenggong Bioengineering Technology Service Co., Ltd. Lipofectamine 2000 transfection reagent was purchased from Invitrogen Inc. Plasmid Purification Kit was purchased from QIAGEN Inc. Ordinary plasmid small kit was purchased from Shanghai Shenggong Bioengineering Technology Service Co., Ltd. MicroRNA microarrays were purchased from Guangzhou Ruibo Biotechnology Co., Ltd. Total RNA extraction reagent TRIzol Reagent was purchased from Invitrogen Inc.; reverse transcription kit—SYBR Premix Ex TaqTM II (DRR081A)—was purchased from Takara Inc. The primers are shown in Table 1.

### 2.4. Cell Proliferation Measured by the MTT Assay

MCF-7 cells that grew in the logarithmic phase with good condition were incubated in a 5% CO_2_, 37°C incubator. Then, baicalin at concentrations of 0 *μ*mol/L (NC), 50 *μ*mol/L, 100 *μ*mol/L, and 150 *μ*mol/L was added separately. After incubating in a 5% CO_2_, 37°C incubator for 24, 48, and 72 hours, the cells were observed under an inverted microscope. Then, the MTT solution was added to terminate the culture, and finally, DMSO was added to each well. The enzyme-linked immunosorbent assay was used to measure the absorbance of each well at OD 490 nm.

### 2.5. Total RNA Extraction and miRNA Microarrays' Screening

Total RNA of MCF-7 cells treated with an optimum concentration of baicalin (experiment group) and the MCF-7 cells not treated with baicalin (control group) was extracted by using TRIzol and miRNA extraction kits according to the manufacturer's instructions, which effectively covered all types of RNA, including miRNA. The nanodrop spectrophotometer was used to measure the quality and quantity of RNA, and the integrity of RNA was measured by gel electrophoresis. miRNA was labeled with the T4RNA ligase labeling method and precipitated with absolute ethanol, and then miRNA microarray hybridization scanning and data analysis were completed. Differential expression genes of breast cancer cells after baicalin intervention were screened by the chip saliency analysis algorithm (SAM 3.11).

### 2.6. Validation of miRNA Expression through Real-Time Fluorescence Quantification of PCR (qRT-PCR)

Total RNA of the blank group and the baicalin group was 500 ng. RNA was mixed with 2 *μ*l dNTP (2.5 mmol/L), 2 *μ*l 10X RT buffer, 0.3 *μ*l RT-specific primer (1 *μ*mol/L), 0.2 *μ*l MMLV reverse transcriptase (200 U/*μ*l), 0.3 *μ*l RNase inhibitor (40 U/*μ*l), and 20 *μ*l RNA enzyme-free water. cDNA was synthesized in the PCR thermal cycler. The data were analyzed by the 2^−ΔΔCT^ method. The experiment was repeated 3 times under the same conditions.

### 2.7. Potential Targeted Genes of MicroRNA Prediction and Enrichment Analysis

The potential targeted genes of miRNA were predicted by miRWalk 3.0 (http://mirwalk.umm.uni-heidelberg.de/) [19, 20]. The DAVID database ver. 6.8 (https://david-d.ncifcrf.gov) was applied for gene ontology (GO) enrichment analysis and pathway enrichment analysis [21].

### 2.8. Statistical Analysis

The data were expressed as *x* ± *s*, and SPSS 22.0 statistical software was used for processing, and the *t*-test was used for comparison of differences between groups. *P* < 0.05 means that the difference was statistically significant.

## 3. Results

### 3.1. Inhibition of Baicalin on the Proliferation of Breast Cancer Cell MCF-7

**Figure 1:**
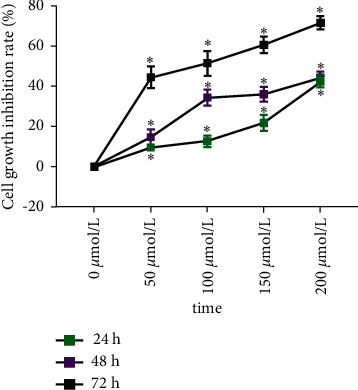
Inhibition of baicalin on the proliferation of breast cancer cell MCF-7 (^*∗*^compared with the control group, *P* < 0.05).

Baicalin at concentrations of 0, 50, 100, 150, and 200 *μ*mol/L was added and interfered with breast cancer cell MCF-7 for 24, 48, and 72 hours. The growth of breast cancer cell MCF-7 was significantly inhibited and was time and concentration dependent within a certain time and concentration range. The differences between the baicalin groups and the negative control group were statistically significant (*P* < 0.05) (Figure 1).

### 3.2. Differentially Expressed MicroRNA

**Figure 2:**
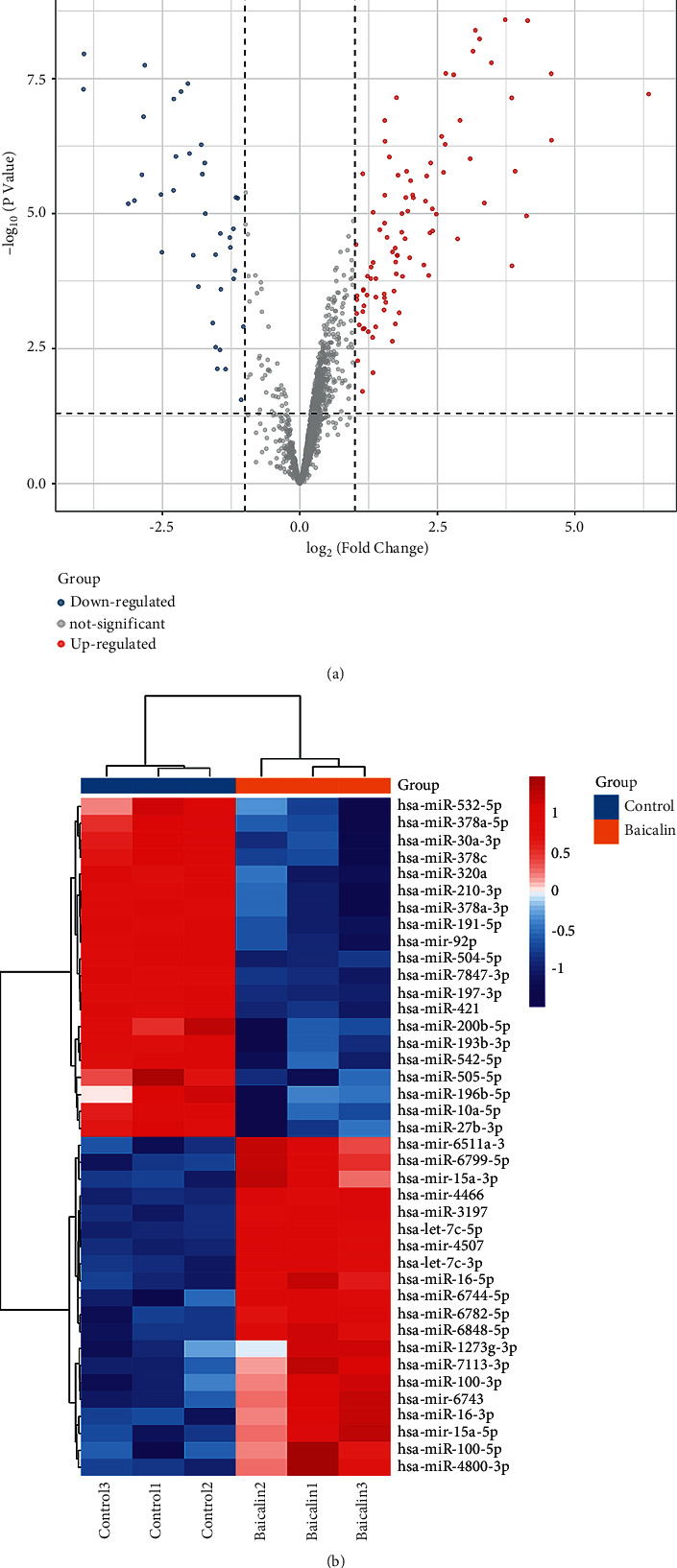
Differentially expressed microRNAs. (a) Volcano map. (b) Clustering heat map.

The concentration of baicalin 150 *μ*mol/L was determined for the subsequent miRNA chip research. Log2FC ≥ 1 or ≤−1 and *P* < 0.05 were used as the standard to screen for differentially expressed microRNA. Finally, a total of 92 upregulated microRNAs and 35 downregulated microRNAs were obtained (Figure 2). The most upregulated miRNAs include hsa-miR-6799-5p, hsa-miR-6126, hsa-miR-4792, hsa-miR-6848-5p, hsa-miR-3197, hsa-miR-6779-5p, and hsa-miR -654-5p. The most downregulated miRNAs include hsa-miR-3911, hsa-miR-504-5p, hsa-miR-30a-3p, hsa-miR-193b-3p, and hsa-miR-181b-5p.

### 3.3. Bioinformatics Analysis of Differentially Expressed MicroRNA

**Figure 3:**
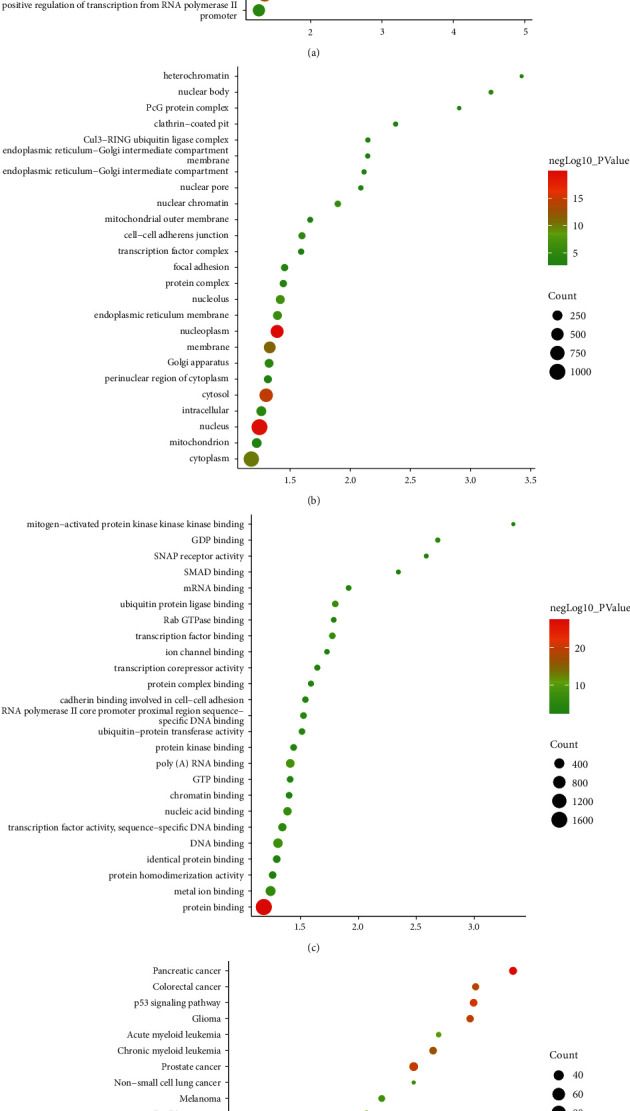
Bioinformatics analysis of differentially expressed microRNAs. (a) Biological processes. (b) Cell components. (c) Molecular functions. (d) Signaling pathways. *X*-axis represents fold enrichment.

miRWalk was utilized to obtain potential mRNA of differentially expressed microRNA. Potential mRNAs were input into DAVID for enrichment analysis (Table S1). The biological processes include transcription, DNA-templated, regulation of transcription, DNA-templated, negative regulation of transcription from RNA polymerase II promoter, positive regulation of transcription, DNA-templated, negative regulation of transcription, DNA-templated, cellular response to hypoxia, protein autophosphorylation, cell-cell adhesion, transcription from RNA polymerase II promoter, cellular response to DNA damage stimulus, and protein ubiquitination (Figure 3(a)). The cell components include the nucleoplasm, nucleus, cytosol, membrane, cytoplasm, nucleolus, endoplasmic reticulum membrane, nuclear chromatin, cell-cell adherens junction, intracellular, nuclear body, Golgi apparatus, protein complex, focal adhesion, and clathrin-coated pit (Figure 3(b)). The molecular functions include protein binding, poly(A) RNA binding, DNA binding, ubiquitin-protein ligase binding, nucleic acid binding, transcription factor binding, metal ion binding, transcription factor activity, sequence-specific DNA binding, GDP binding, RNA polymerase II core promoter proximal region sequence-specific DNA binding, mRNA binding, and ubiquitin-protein transferase activity (Figure 3(c)). The signaling pathways include pancreatic cancer, endocytosis, pathways in cancer, p53 signaling pathway, prostate cancer, glioma, colorectal cancer, Hippo signaling pathway, FoxO signaling pathway, chronic myeloid leukemia, and signaling pathways regulating pluripotency of stem cells (Figure 3(d)).

### 3.4. Validation of hsa-miR-15a, hsa-miR-100, hsa-miR-16, and hsa-let-7c in the Microarrays' Results Using qRT-PCR

**Figure 4:**
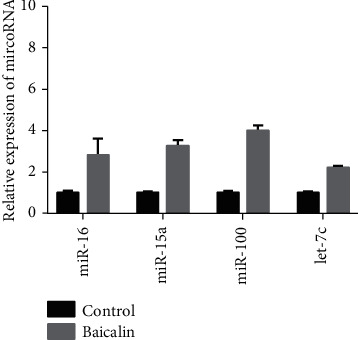
Validation of hsa-miR-15a, hsa-miR-100, hsa-miR-16, and hsa-let-7c in the microarrays' results using qRT-PCR.

The PCR electropherogram showed that the internal reference and miRNA bands were clearly visible, indicating that the extracted total RNA was relatively intact and substantially free of degradation. The UV spectrophotometer showed that the absorbance values of the samples at 260–280 nm were between 1.8 and 2.0, indicating that the purity meets the requirements. Then, qPCR was utilized to detect the expression of hsa-miR-15a, hsa-miR-100, hsa-miR-16, and hsa-let-7c miRNAs. The results showed that the expression of hsa-miR-15a, hsa-miR-100, hsa-miR-16, and hsa-let-7c was upregulated (*P* < 0.05). This was consistent with the results of the miRNA microarray (Figure 4).

## 4. Discussion

MicroRNA (or miRNA) is a small noncoding RNA molecule ranging in length from 20 to 25 nucleotides that binds primarily to the 3′ untranslated region (UTR) of messenger RNA, resulting in downregulation of the target protein by degradation or translational inhibition of mRNA [22]. miRNAs are not directly encoded by their corresponding genes. RNA polymerase II in the nucleus can transcribe miRNA genes to form PRI-miRNAs. Next, PRI-miRNA is further cleaved by a complex of Drosha enzyme and Pasha/DGCR8 protein into precursor miRNA (pre-miRNA) containing approximately 70 nucleotides [23]. The transporter Exportin-5 cleaves pre-miRNA into a miRNA duplex containing approximately 22 nucleotides, with the mature single-stranded miRNA retained and the other single-stranded RNA degraded [24, 25]. The total number of miRNAs in human genes is expected to exceed one thousand and regulate approximately 30% of human genes. In vivo, miRNA regulates the expression of its target genes mainly by inducing degradation of target mRNA and inhibiting translation of target mRNA [26]. In breast cancer cells, miRNAs can bind to the mRNA 3′-UTR of a specific gene through complementary pairing, resulting in inhibition of translation of the target gene, thereby regulating gene expression and function. As an endogenous inhibitor of gene expression, miRNA forms a complex intracellular network with its upstream and downstream effector molecules, regulating the process of cell life activities, such as the occurrence and development of tumor cells [27, 28].

Recent studies have shown that dysregulation of miRNAs not only affects cellular processes associated with carcinogenesis, tumor resistance, invasion, and metastasis but may also have a direct impact on the effectiveness of treatment [29, 30]. Among them, there are oncogenic miRNA and tumor suppressor miRNA, which act on tumor suppressor genes and proto-oncogenes, respectively. These miRNAs regulate the progression of breast cancer in drug metabolism enzymes, breast cancer stem cells, and single-nucleotide polymorphisms in breast cancer [31]. For example, compared with normal breast tissue, the expression of miR-21 and miR-155 was upregulated, and that of miR-10b, miR-125b, and miR-145 was downregulated in breast cancer tissues [32]. Volinia et al. found that miR-17-5p, miR-20a, miR-21, miR-106a, and miR-155 were closely related to tumorigenesis in tumor tissues such as breast cancer [33]. At present, the research found that proto-oncogene miRNAs closely related to breast cancer are miR-10b, miR-21, miR-155, miR-181a/181b, and so on [34], while tumor suppressor miRNAs include the lethal-7 family (Let-7), miR-125a/125b, miR-206, and miR-34 [35].

miRNAs also play an important role in the regulation of breast cancer stem cells. Shimono et al. showed a decrease in the expression of the miR-200 family (miR-200a/-200b/-200c, miR-101, miR-182miR-183, and miR-96) in CD44+CD24-/low lineage-primary breast cancer stem cells [36]. miR-200c also strongly inhibits the formation of mammary ducts and the ability of breast cancer stem cells to form tumors in normal breast stem cells [33]. In the cancer cells of breast cancer with bone metastasis and lung metastasis, the expression of 8 miRNAs decreased, including miR-126, miR-206, and miR-335; these miRNAs also decreased in distant metastatic cancer cells in other sites [37, 38]. MicroRNAs also begin to play a role in early diagnosis and typing of breast cancer [39, 40], such as luminal type A (upregulated expression: let-7a/b/c/f, miR-10a, -30a-3p, -30a-5p, -99a, -100, -126, -130a, -136, -145, -199a/b, -224, -214, and -342) and luminal type B (upregulated expression: let-7b/c/f, miR-10a, -30a-3p, -30a-5p, -93, -25, -106a/b, -224, and -342).

Recent studies indicate that miRNAs are key regulators of these drug transporters, such as miRNAs targeting MDR1 including miR-451 [41], miR-7, miR-345 [42], and miR-326 [43]. Increased expression of miRNAs associated with response to radiation therapy results in increased resistance of the tumor to treatment. Many miRNAs that have been found to be involved in drug resistance of breast cancer include the miRNA-let-7 family, miR-451, miR-21, miR-101, and miR-221/222 [44, 45]. miRNAs associated with resistance to endocrine therapy include miR-221, -222, and -181b, miR-30c, and miR-301 [46, 47].

In summary, miRNA dysregulation in breast cancer, whether through polymorphisms in miRNA sequences, binding sites in target genes, or epigenetic mechanisms, has been shown to play a key role in the entire carcinogenesis process. Meanwhile, this study shows that baicalin can interfere with these miRNAs, suggesting that baicalin may exert antitumor effects on breast cancer at miRNA levels.

## 5. Conclusion

Baicalin inhibits the proliferation of breast cancer cells. It may achieve antitumor effects through regulating microRNAs so as to affect the DNA replication (such as cellular response to DNA damage stimulus and DNA binding), RNA transcription (such as regulation of transcription, DNA-templated, transcription from RNA polymerase II promoter, and transcription factor binding), protein synthesis (such as mRNA binding, Golgi apparatus, and protein complex), endocytosis, pathways in cancer, p53 signaling pathway, and so on.

## Figures and Tables

**Table 1:** Primers.

miRNA	Forward primer	Reverse primer
miR-15a	5′-GGGTAGCAGCACATAATGG-3′	5′-CAGTGCGTGTCGTGGAGT-3′
miR-100	5′-GCTCTGAACCGTAGATCCGAAC-3′	5′-GTGCAGGGTCCGAGGT-3′
miR-16	5′-GGGTAGCAGCACGTAAATA-3′	5′-CAGTGCGTGTCGTGGAGT-3′
miR-let-7c	5′-GCCGCTGAGGTAGTAGGTTGTAT-3′	5′-GTGCAGGGTCCGAGGT-3′
U6	5′-CTCGCTTCGGCAGCACATATACT-3′	5′-ACGCTTCACGAATTT-GCGTGTC-3′

## Data Availability

The data that support the findings of this study are openly available in supplementary materials.

## Supplementary Materials

Supplementary Materials Table S1: bioinformatics analysis of differentially expressed microRNAs.
