# Supplementary material for: Exploring the Mechanism of Baicalin Intervention in Breast Cancer Based on MicroRNA Microarrays and Bioinformatics Strategies
Source: Evid Based Complement Alternat Med. 2021 Dec 20;2021:7624415. doi: 10.1155/2021/7624415 (PMC8712139; doi:10.1155/2021/7624415)
Supplement: Supplementary Materials — Table S1: bioinformatics analysis of differentially expressed microRNAs. [file 7624415.f1.pdf]

Table S1 Bioinformatics Analysis of Differential expressed microRNA

Category	Term	Description	Count	%	PValue	Genes	Fold Enric
	GO:0006351	transcription, DNA-tem	424	14.40217	2.49E-13	ZNF175, N	1.37221
	GO:0006355	regulation of transcripti	323	10.97147	1.04E-09	ZNF175, B	1.358802
	GO:0000122	negative regulation of tr	165	5.60462	3.52E-07	WWC1, U	1.44995
	GO:0045893	positive regulation of tr	125	4.245924	4.86E-07	RORA, ET	1.535693
	GO:0045892	negative regulation of tr	120	4.076087	1.39E-06	RBPJ, PHE	1.521536
	GO:0071456	cellular response to hyp	34	1.154891	5.76E-06	ERO1A, A	2.240831
	GO:0046777	protein autophosphoryla	50	1.69837	1.67E-05	DDR1, SM	1.83926
	GO:0098609	cell-cell adhesion	70	2.377717	2.50E-05	MRE11, D	1.634294
	GO:0006366	transcription from RNA	117	3.974185	2.60E-05	CCNT1, N	1.443012
	GO:0006974	cellular response to DN	56	1.902174	5.35E-05	CDKN1A,	1.703437
	GO:0016567	protein ubiquitination	86	2.921196	5.64E-05	TRIM72, U	1.515673
	GO:0031647	regulation of protein sta	25	0.849185	1.09E-04	KHDRBS1	2.259662
	GO:0045944	positive regulation of tr	198	6.725543	1.33E-04	EIF4A3, SI	1.27702
	GO:0070534	protein K63-linked ubiq	16	0.543478	2.66E-04	UBE2D4, U	2.736023
	GO:0016055	Wnt signaling pathway	49	1.664402	3.28E-04	DRAXIN,	1.657891
	GO:0017148	negative regulation of tr	21	0.713315	3.66E-04	NANOS1,	2.29083
	GO:0018107	peptidyl-threonine phos	16	0.543478	3.76E-04	PDPK1, D	2.664023
	GO:0043065	positive regulation of ap	71	2.411685	3.83E-04	MTCH2, C	1.497403
	GO:0008284	positive regulation of ce	102	3.464674	4.34E-04	KLB, VIPF	1.384892
	GO:0050821	protein stabilization	38	1.290761	4.64E-04	CDKN1A,	1.767853
	GO:0030511	positive regulation of tr	12	0.407609	5.03E-04	TGFBR3,	3.163527
	GO:0007223	Wnt signaling pathway,	16	0.543478	5.23E-04	TCF7L2, F	2.595714
	GO:0008285	negative regulation of c	88	2.98913	6.71E-04	DDR1, CD	1.406012
	GO:0010863	positive regulation of ph	7	0.237772	8.42E-04	PDGFRA,	4.921042
ogical proce	GO:0007264	small GTPase mediated	59	2.004076	8.87E-04	RAB3B, A	1.517464
	GO:0005654	nucleoplasm	593	20.14266	1.15E-20	ZNF175, H	1.392811
	GO:0005634	nucleus	1032	35.05435	4.46E-20	RBPJ, NO	1.246201
	GO:0005829	cytosol	660	22.41848	1.68E-15	USP6NL, F	1.301869
	GO:0016020	membrane	448	15.21739	8.06E-12	PI4K2B, T	1.331564
	GO:0005737	cytoplasm	941	31.96332	1.12E-10	RBPJ, NO	1.17831
	GO:0005730	nucleolus	186	6.317935	3.23E-07	SRP19, MI	1.419185
	GO:0005789	endoplasmic reticulum	184	6.25	1.20E-06	ERO1A, F	1.395781
	GO:0000790	nuclear chromatin	56	1.902174	1.89E-06	LDB1, NU	1.897306
	GO:0005913	cell-cell adherens juncti	79	2.683424	1.78E-05	MRE11, D	1.599305
	GO:0005622	intracellular	257	8.72962	3.22E-05	ZNF175, C	1.261641
	GO:0016604	nuclear body	16	0.543478	3.72E-05	ZNF496, Z	3.170391
	GO:0005794	Golgi apparatus	175	5.944293	4.97E-05	UBXN2B,	1.325971
	GO:0043234	protein complex	91	3.091033	2.22E-04	CDKN1A,	1.444278
	GO:0005925	focal adhesion	87	2.955163	2.39E-04	ARF1, FLT	1.454954
	GO:0005905	clathrin-coated pit	20	0.679348	3.24E-04	CUBN, TF	2.377793
	GO:0005739	mitochondrion	249	8.45788	3.28E-04	TDRKH, IS	1.223286
	GO:0000792	heterochromatin	11	0.373641	4.76E-04	CBX8, ME	3.425154
	GO:0031463	Cul3-RING ubiquitin lig	22	0.747283	7.18E-04	KLHL36, F	2.147112
	GO:0005643	nuclear pore	23	0.78125	7.98E-04	EIF5A, EIF	2.088825
	GO:0048471	perinuclear region of cy	125	4.245924	8.38E-04	RAB3B, P	1.31621
	GO:0005793	endoplasmic reticulum-	22	0.747283	8.92E-04	TRAPPC2,	2.115536
	GO:0033116	endoplasmic reticulum-	21	0.713315	9.93E-04	MCFD2, S	2.145587
	GO:0005667	transcription factor com	47	1.596467	0.001159	RBPJL, LD	1.592382
	GO:0031519	PcG protein complex	12	0.407609	0.00126	CBX8, YY	2.906191
ll compone	GO:0005741	mitochondrial outer me	38	1.290761	0.001556	MTCH2, D	1.667647
	GO:0005515	protein binding	1651	56.08016	3.20E-28	TFRC, ITS	1.184658

	GO:0044822	poly(A) RNA binding	253	8.59375	2.34E-09	TFRC, EIF	1.412584
	GO:0003677	DNA binding	347	11.78668	2.33E-08	ZNF175, H	1.306657
	GO:0031625	ubiquitin protein ligase l	82	2.785326	6.43E-08	GPI, CDKN	1.801024
	GO:0003676	nucleic acid binding	217	7.370924	1.53E-07	ZNF175, E	1.388709
	GO:0008134	transcription factor bind	80	2.717391	1.81E-07	DDX3X, C	1.775658
	GO:0046872	metal ion binding	408	13.8587	4.47E-07	ZNF175, Z	1.243046
	GO:0003700	transcription factor activ	205	6.963315	3.63E-06	ZNF175, IF	1.344677
	GO:0019003	GDP binding	23	0.78125	1.02E-05	RAB3B, PI	2.68486
	GO:0000978	RNA polymerase II core	86	2.921196	4.23E-05	ZNF253, G	1.527066
	GO:0003729	mRNA binding	38	1.290761	7.77E-05	FYTTD1, F	1.91629
	GO:0004842	ubiquitin-protein transfe	79	2.683424	1.21E-04	TRIM71, U	1.513627
	GO:0098641	cadherin binding involv	71	2.411685	1.49E-04	MRE11, D	1.543291
	GO:0003714	transcription corepresso	53	1.800272	2.21E-04	KDM5B, L	1.645764
	GO:0019901	protein kinase binding	86	2.921196	3.37E-04	USP37, HS	1.441777
	GO:0017137	Rab GTPase binding	38	1.290761	3.65E-04	USP6NL, C	1.787584
	GO:0042802	identical protein binding	154	5.230978	4.09E-04	TFRC, AR	1.296064
	GO:0005484	SNAP receptor activity	16	0.543478	5.44E-04	STX17, ST	2.586086
	GO:0032403	protein complex binding	52	1.766304	5.98E-04	CDKN1A,	1.591196
	GO:0005525	GTP binding	86	2.921196	6.77E-04	RAB3B, C	1.41174
	GO:0003682	chromatin binding	87	2.955163	7.79E-04	CCNT1, LI	1.402588
	GO:0046332	SMAD binding	16	0.543478	0.001754	SMAD2, H	2.34552
	GO:0042803	protein homodimerizatic	146	4.959239	0.001848	SLC27A1,	1.260717
	GO:0044325	ion channel binding	31	1.052989	0.00244	HSP90AB1	1.729302
lecular func	GO:0031435	mitogen-activated protei	9	0.305707	0.002495	MAP2K4,	3.337192
	hsa05212	Pancreatic cancer	29	0.985054	1.57E-07	PIK3CD, T	2.802824
	hsa04144	Endocytosis	69	2.34375	5.94E-07	ARF1, TFF	1.798636
	hsa05200	Pathways in cancer	100	3.396739	6.89E-07	ETS1, FGF	1.598522
	hsa04115	p53 signaling pathway	28	0.951087	1.31E-06	CDKN1A,	2.625394
	hsa05215	Prostate cancer	33	1.120924	2.09E-06	CDKN1A,	2.355822
	hsa05214	Glioma	27	0.91712	2.42E-06	CDKN1A,	2.609526
	hsa05210	Colorectal cancer	26	0.883152	3.18E-06	LEF1, PIK	2.634468
	hsa04390	Hippo signaling pathwa	47	1.596467	4.44E-06	PATJ, BM	1.955384
	hsa04068	FoxO signaling pathway	43	1.460598	5.20E-06	CDKN1A,	2.015927
	hsa05220	Chronic myeloid leuken	28	0.951087	6.67E-06	CDKN1A,	2.443075
	hsa04550	Signaling pathways regu	43	1.460598	1.75E-05	BMPR2, W	1.92953
	hsa05205	Proteoglycans in cancer	55	1.868207	3.42E-05	CDKN1A,	1.727603
	hsa05221	Acute myeloid leukemia	22	0.747283	6.99E-05	TCF7L2, Z	2.468004
	hsa05222	Small cell lung cancer	29	0.985054	7.21E-05	PTEN, XIA	2.143336
	hsa05161	Hepatitis B	42	1.42663	9.86E-05	CDKN1A,	1.819669
	hsa05166	HTLV-I infection	64	2.173913	1.26E-04	CDKN1A,	1.582914
	hsa05218	Melanoma	25	0.849185	1.48E-04	CDKN1A,	2.212039
	hsa05223	Non-small cell lung can	21	0.713315	2.25E-04	CDKN2A,	2.355822
	hsa05169	Epstein-Barr virus infec	35	1.188859	5.07E-04	RBPJL, CD	1.802268
	hsa05160	Hepatitis C	37	1.256793	6.42E-04	CDKN1A,	1.747677
	hsa04110	Cell cycle	35	1.188859	6.98E-04	CDKN1A,	1.773199
	hsa04012	ErbB signaling pathway	27	0.91712	7.24E-04	CDKN1A,	1.949646
	hsa04722	Neurotrophin signaling	34	1.154891	7.87E-04	CAMK2A,	1.779954
	hsa04350	TGF-beta signaling path	26	0.883152	9.72E-04	BMPR2, A	1.944488
naling pathv	hsa04520	Adherens junction	23	0.78125	0.001068	SMAD2, T	2.035076

Bonferroni
1.50E-09
6.27E-06
0.002119
0.002929
0.008348
0.034159
0.095724
0.139911
0.145391
0.275689
0.288669
0.482911
0.552532
0.799344
0.862127
0.890065
0.896611
0.901068
0.926958
0.939126
0.952014
0.957317
0.982603
0.993809
0.995276
1.08E-17
4.20E-17
1.57E-12
7.60E-09
1.06E-07
3.04E-04
0.001128
0.00178
0.016682
0.029918
0.034514
0.045822
0.189204
0.202007
0.263383
0.266265
0.361758
0.492151
0.528953
0.546445
0.568785
0.608101
0.66514
0.695567
0.769749
6.07E-25

4.43E-06
4.43E-05
1.22E-04
2.91E-04
3.43E-04
8.49E-04
0.006868
0.019243
0.077194
0.137157
0.205291
0.246565
0.342188
0.472394
0.500237
0.539744
0.644404
0.679017
0.723902
0.77245
0.96434
0.970195
0.990334
0.991299

4.55E-05
1.72E-04
1.99E-04
3.78E-04
6.03E-04
7.00E-04
9.19E-04
0.001283
0.001502
0.001927
0.005034
0.009842
0.020007
0.020623
0.028082
0.035863
0.041952
0.063035
0.136214
0.169275
0.182839
0.188792
0.203447
0.244908
0.265777
